# Sustained xanthine oxidase inhibitor treat to target urate lowering therapy rewires a tight inflammation serum protein interactome

**DOI:** 10.21203/rs.3.rs-3770277/v1

**Published:** 2024-01-02

**Authors:** Concepcion Sanchez, Anamika Campeau, Ru Liu-Bryan, Ted Mikuls, James O’Dell, David Gonzalez, Robert Terkeltaub

**Affiliations:** University of California San Diego; University of California San Diego; University of California San Diego; University of Nebraska Medical Center; University of Nebraska Medical Center; University of California San Diego; University of California San Diego

**Keywords:** Xanthine oxidase, allopurinol, febuxostat, gout, inflammation, proteomics, Complement, C8, TGFbeta

## Abstract

**Background:**

Effective xanthine oxidoreductase inhibition (XOI) urate-lowering treatment (ULT) to target significantly reduces gout flare burden and synovitis between 1–2 years therapy, without clearing all monosodium urate crystal deposits. Paradoxically, treat to target ULT is associated with increased flare activity for at least 1 year in duration on average, before gout flare burden decreases. Since XOI has anti-inflammatory effects, we tested for biomarkers of sustained, effective ULT that alters gouty inflammation.

**Methods:**

We characterized the proteome of febuxostat-treated murine bone marrow macrophages. Blood samples (baseline and 48 weeks ULT) were analyzed by unbiased proteomics in febuxostat and allopurinol ULT responders from two, independent, racially and ethnically distinct comparative effectiveness trial cohorts (n=19, n=30). STRING-db and multivariate analyses supplemented determinations of significantly altered proteins via Wilcoxon matched pairs signed rank testing.

**Results:**

The proteome of cultured IL-1b-stimulated macrophages revealed febuxostat-induced anti-inflammatory changes, including for classical and alternative pathway complement activation pathways. At 48 weeks ULT, with altered purine metabolism confirmed by serum metabolomics, serum urate dropped >30%, to normal (<6.8 mg/dL) in all the studied patients. Overall, flares declined from baseline. Treated gout patient sera and peripheral blood mononuclear cells (PBMCs) showed significantly altered proteins (p<0.05) in clustering and proteome networks. CRP was not a useful therapy response biomarker. By comparison, significant serum proteome changes included decreased complement C8 heterotrimer C8A and C8G chains essential for C5b-9 membrane attack complex assembly and function; increase in the NLRP3 inflammasome activation promoter vimentin; increased urate crystal phagocytosis inhibitor sCD44; increased gouty inflammation pro-resolving mediator TGFB1; decreased phagocyte-recruiting chemokine PPBP/CXCL7, and increased monocyte/macrophage-expressed keratin-related proteins (KRT9,14,16) further validated by PBMC proteomics. STRING-db analyses of significantly altered serum proteins from both cohorts revealed a tight interactome network including central mediators of gouty inflammation (eg, IL-1B, CXCL8, IL6, C5).

**Conclusions:**

Rewiring of inflammation mediators in a tight serum protein interactome was a biomarker of sustained XOI-based ULT that effectively reduced serum urate and gout flares. Monitoring of the serum and PBMC proteome, including for changes in the complement pathway could help determine onset and targets of anti-inflammatory changes in response to effective, sustained XOI-based ULT.

Trial Registration: ClinicalTrials.gov Identifier: NCT02579096

## Introduction

Gout is characterized by acute arthritis flares that typically are excruciatingly painful and incapacitating (1, 2). Exogenous factors, including joint trauma, certain dietary excesses, and alcohol consumption, can trigger flares (3–5). Gout flares require treatment with NSAIDs, corticosteroids, and colchicine, which are nonselective, frequently toxic, and interact frequently with other medications (1, 6, 7). Undertreated, gout commonly progresses to more frequent flares, chronic arthritis, and permanent joint damage (1). Gout also is linked to prevalent comorbidities mediated by low-grade inflammation (eg, obesity, type 2 diabetes, atherosclerosis)(1, 8).

Treatment of hyperuricemia with XOI drugs (principally by using allopurinol or febuxostat) is central to gout management (6, 7). However, effective XOI urate-lowering treatment (ULT) to target also paradoxically induces an elevated gout flare burden early in treatment (6, 7, 9); remodeling of articular monosodium urate (MSU) crystal deposits and consequent release of free crystals are held partly responsible (10–12). Notably, changes in a subset of CD14 positive monocytes, overactivation of CD8 + T cells, and upregulate arachidonate metabolism also have been implicated perpetuating systemic gouty inflammation after ULT initiation (13).

MSU crystals stimulate gouty inflammation in large part by activating monocytes and macrophages, promoting NLRP3 inflammasome-mediated IL-1b release, and neutrophil influx and activation that amplify the inflammatory cascade (1, 14). C5 cleavage on the MSU crystal surface, and consequent C5b-9 complement membrane attack complex (MAC) assembly and membrane pore-forming activity play a major role in the model gouty arthritis inflammatory process (15, 16).

Recent clinical trials have demonstrated that effective XOI urate-lowering treatment (ULT) to target eventually reduces gout flare burden and synovitis between 1–2 years therapy (17–19). Importantly, flares decrease in this time frame despite total resolution of urate crystal deposits being far slower, and particularly difficult to achieve (10), and continuing systemic inflammation even in the periods between flares and in clinical remission (13). In clinical practice, this situation is associated with lack of clarity on how long anti-inflammatory gout flare prophylaxis, typically using low dose colchicine, is necessary after initiating XOI-based ULT and achieving the serum urate target (9).

Significantly, XOI drugs exert anti-inflammatory effects in monocytes and some other cells, including by antioxidant and urate-lowering effects (20–24). For example, XOI drugs inhibit NLRP3 inflammasome activation, IL-1b release, and chemokine expression in cultured monocyte/macrophage lineage cells (20, 21). *In vivo*, XOI drugs limit mouse models of atherosclerosis, nonalcoholic steatohepatosis, and certain other diseases involving low-grade chronic inflammation and oxidative stress processes (20–24). Hence, we conducted a seminal study to test the hypothesis that sustained, effective XOI-based ULT re-programs inflammatory networks in gout by 48 weeks therapy, and that this could be detectable using unbiased proteomics.

The data revealed the ability of proteomics to detect anti-inflammatory changes in cultured XOI-treated macrophages, and in response to sustained, effective XOI-based ULT in gout patient sera and PBMCs. Our results provide unbiased evidence that sustained XOI treat to target ULT in gout re-wires complement activation and other inflammatory pathways.

## Methods

### Subjects

As previously reported in detail (25), Cohort 1 human subjects were studied under informed consent, and with local IRB approval (at the Jennifer Moreno San Diego Veterans Affairs Medical Center) in a prospective study ancillary to the national, multi-site comparative effectiveness ULT trial VA CSP594 STOP GOUT (26). In that trial, gout patients were randomized to a treat to urate target ULT regimen using allopurinol or the more selective XOI febuxostat (23). Unless contraindicated, colchicine was prescribed as the primary anti-inflammatory gout flare prophylaxis, with colchicine routinely stopped at 6 months ULT. Twenty consecutive patients meeting the 2015 ACR/EULAR gout classification criteria (27), and with current hyperuricemia, were recruited from the Rheumatology Outpatient Clinic at the San Diego site (25). Once again (25), the gout validation cohort (Cohort 2, n = 30)) was from the University of Nebraska Medical Center, in Omaha, NE research site, under informed consent and with local IRB approval. We previously characterized Cohort 1 gout patient metabolomic profiles at time zero and 12 and 24 weeks of treat to target ULT, done in a blinded way for the XOI used, and following the trial protocol (25).

### Proteomics:

Sera were obtained from both cohorts, with peripheral blood mononuclear cells (PBMCs) also prepared from Cohort 1 samples. All subjects were clinically assessed by study physicians for palpable tophaceous disease and presence of active flare or quiescent arthritis, with co-morbidities and current medications also recorded.

For serum collection, research personnel collected non-fasting blood samples into 10 ml BD Vacutainer Blood Collection Tubes containing spray-coated silica and a polymer gel to facilitate serum separation. Following 30 min incubation at room temperature, tubes were centrifuged for 10 min at 2000×g and sera were transferred into 1.7ml tubes and immediately frozen and stored at − 80°C until analyses were performed.

For PBMC preparation, non-fasting blood samples collected into 10 ml BD Vacutainer K2 EDTA Plus Blood Collection Tubes were transferred to a conical tube containing equal volume of PBS (~ total 20 ml). The samples were then layered over Sigma Histopaque^®^-1077 (20 mL) in 50 mL conical tubes at room temperature, followed by centrifugation at 400×g in a swinging bucket centrifuge for 30 minutes at room temperature with no brake. The white cellular layer containing PBMCs at the interface between the plasma and density gradient was collected and washed in PBS by dilution and centrifugation for 10 minutes at 250×g. PBMC pellets were immediately frozen and stored at − 80°C until analyzed.

### Mass Spectrometry Proteomics:

Sample preparation for proteomic analyses of BMDMs and patient sera was done as we previously described in extensive detail (28), with slight modification to the sample digestion protocol, which used 10μg trypsin in 50mM TEAB at 47°C for 3 hours. After protein extraction and trypsin digest, 50ug aliquots of samples were reserved for TMT pro-labeling (28). Bridge channels for downstream data analysis of serum samples, were prepped by combining 5μg of all samples; 50μg aliquots of our bridge sample were then prepared for each TMT-plex (5 total).

### Mass spectrometry data acquisition

Serum and BMDM proteomic data were acquired as described in detail (28). In brief, serum and BMDM proteomic data were acquired through an Thermo Orbitrap Fusion equipped with a Thermoeasy nLC 1000. For Mass spectrometry data search, raw mass spectrometry files were searched using Proteome Discoverer 2.5.0.400. The SEQUEST algorithm was used for spectral matches of raw data with *in silico* generated protein databases. Serum samples were searched against the UniProt *Homo sapiens* proteome (05-06-2023) and BMDM samples were searched against the *Mus musculus* proteome (05-06-23).

### Mass Spectrometry Metabolomics

Sample preparation of patient sera for metabolomics were essentially as previously described (28). In brief, for data Analysis, metabolite features were first normalized to the intensity of value of the internal standard, sulfamethazine, in each sample and then multiplied by 1E6. Missing values (with peak intensities of 0) in metabolite features were set to NA. Then, features with more than 20% missing values per group (timepoint) were removed from analysis. Missing values in remaining features were imputed using K-Nearest Neighbor (KNN) imputation using the *impute* R package (1.68.0). Intensity values were then log2 transformed.

Principal coordinate analysis (PcoA) was conducted with metabolite features, using Bray-Curtis distance calculation in the *stats* R package. PERMANOVA analysis was conducted using categorical metadata and metabolite features using Bray-Curtis distance calculation in the ADONIS R package. Binary comparisons between timepoints were done through the R *stats* package using Students T-test. Volcano plots were created in GraphPad Prism. All other plots were made using *ggplot* package in R. MetaboAnalyst (5.0) was used for metabolite functional enrichment analysis using MS peaks ranked by Student’s T test p-values. A p-value cutoff of 0.05 was used for the mummichog algorithm.

### Murine Bone Marrow Derived Macrophage (BMDM) isolation:

Bone marrow cells were isolated from 12-week-old C57BL/6 mice and were then cultured in RPMI containing 10% FBS, penicillin (100 U/ml), streptomycin (100μg/ml) and 20% L929 conditioned media *in vitro* for 7 days to generate BMDMs.

### Statistical analyses:

Paired statistical analyses of gout patient serum and PBMC samples across two timepoints (UCSD cohort), and for three timepoints for sera (Nebraska cohort), were conducted to identify significantly altered proteins. Unpaired statistical analyses were conducted for the cultured mouse BMDM samples. Significantly altered proteins were calculated using a Wilcoxon matched pairs signed rank test using Graphpad Prism with a *p*-value cutoff of 0.1 (serum) or 0.05 (BMDM, PBMC).

For multivariate Analysis, Principal Component Analysis (PCA) was conducted using the *stats* R package using all normalized protein features. Principal Coordinate Analysis (PCoA) was conducted using the *stats* R package using the Euclidean Distance Matrix (EDM) of normalized protein features. PERMANOVA analysis was used to calculate data influence by metadata categories.

Gene Ontology enrichment analysis was conducted through input of significantly altered proteins in both diseases to their respective controls into Cytoscape. Protein interactome analysis was conducted through input of significantly altered proteins in both diseases to their respective controls into String-DB with an interaction confidence of 0.700 (high-confidence).

## Results

### Effects of Febuxostat on BMDMs i n vitro

**Figure 1:**
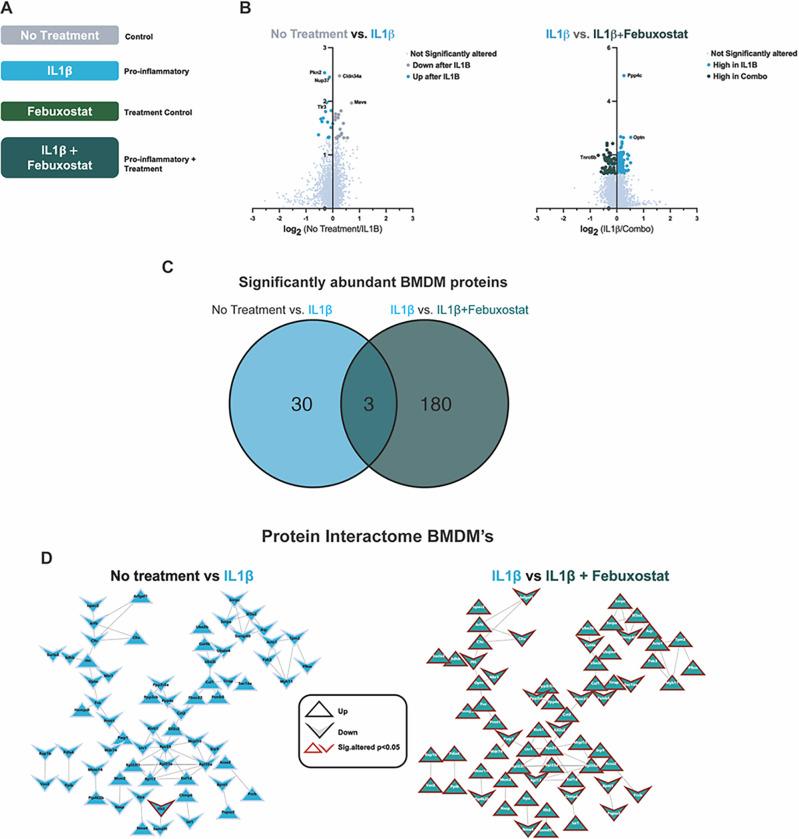
Bone Marrow Derived Macrophage (BMDMs) Proteomics. A. BMDM treatment schematic B. Volcano plots of log2-fold change relative protein abundance versus log10 p-value. Points are colored by condition they are found higher in and sized by p-value significance (p-value<0.05, Wilcoxon signed rank test). C. Venn Diagram displaying overlap of differentially abundant proteins in IL1β and IL1β+Febuxostat treated macrophages. D. Protein interactome from String-DB using significantly altered proteins in respective binary comparison of BMDM treatments. Nodes are shaped based on the direction of relative abundance change after respective treatments and outlined in red if found to be significantly altered (p-value<0.05)

We incubated BMDMs with IL-1β to model the gout pro-inflammatory state (4, 14)(Fig. 1A)(4, 14). Cells were treated with and without the selective XOI febuxostat, since allopurinol non-selectively inhibits both purine and pyrimidine metabolism (29). We first identified significantly altered proteins between untreated and IL-1β-treated macrophages (mock gouty inflammation group) *in vitro*, with 32 proteins found to be significantly altered in response to IL-1b (Fig. 1B, left). Next, we compared IL-1β-treated macrophages with febuxostat co-treated macrophages, which demonstrated suppression of multiple pro-inflammatory proteome changes triggered by IL-1β. Specifically, we found 184 significantly altered (p < 0.05) proteins (Fig. 1B, right), of which 71 proteins were found to interact via STRING-DB analysis (confidence = 0.700) (Fig. 1C, right).

### Effects of XOI-based ULT to target in gout patients

#### Validation of XOI treatment effects on purine metabolism and the serum metabolome

We previously validated XOI treatment effects on purine metabolism in Cohort 1 (25). Here, we conducted untargeted metabolomics on sera of gout patients on effective serum treat to target ULT in Cohort 2 subjects treated with either febuxostat or allopurinol for 48 weeks. We annotated metabolite features using the Global Natural Products Social Molecular Networking (GNPS) platform. Since timepoint significantly influenced our paired proteomic data set, we conducted paired binary comparisons between timepoints. Comparison of baseline (BL) and proteomics endpoint 48wks of ULT revealed several significantly altered metabolites, with some significantly changed by 24wks ULT (Supplemental Fig. 2A). Functional enrichment analysis of all identified metabolite features, using MS1 peak information, validated serum metabolome changes in purine and pyrimidine metabolism in Cohort 2 in this study. These findings were associated with significant changes in multiple other pathways, including arachidonic acid metabolism, and most pronounced for linoleate metabolism at 24 and 48wks ULT (Supplemental Fig. 1B). The new findings for Cohort 2 reinforced previously published effects of XOI treatment on the serum metabolome and lipidome in gout patients of Cohort 1 and on the serum lipidome in both Cohorts 1 and 2 (25).

#### Effects of XOI treatment to urate target on the serum proteome

**Figure 2:**
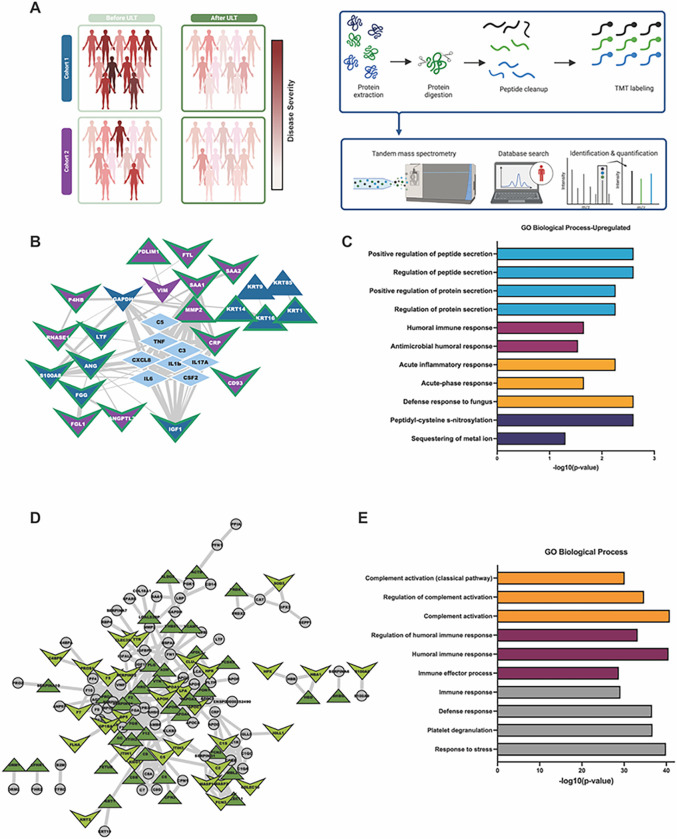
Patient Serum Proteomics. A. Experimental design for proteomics patient cohorts. Cohort 1= UCSD Cohort, Cohort 2= Nebraska cohort B. Protein interactome from String-DB using significantly altered proteins identified in each cohort indecently along with central gout mediators. Nodes are colored by cohort they were found to be significantly altered in and shaped by their direction of change after treatment with ULT. Edges are sized by strength of interaction. C. Gene ontology enrichment analysis of significantly altered proteins from both proteomic cohorts. Enrichment was conducted on Cytoscape with the Human Proteome as background. D. Protein interactome of the detected overlapping proteins from both cohorts. Nodes are colored based on whether their abundance change was the same in both cohorts after 48wks of ULT, and shaped based on their direction of change after ULT. E. Gene ontology enrichment analysis of overlapping proteins from both cohorts. Enrichment was conducted on Cytoscape with the Human Proteome as background.

Global serum proteome changes before and at 48wks XOI-based ULT were found in gout patient Cohort 1 and the independent validation Cohort 2 (Fig. 2B), whose demographics and changes in serum urate are summarized (Fig. 2A, Supplemental Fig. 1A & 1B). We observed overall decrease in serum urate (sUA) levels after 48wks ULT, but relatively stable C-reactive protein (CRP) levels after ULT in both cohorts. Examining each cohort independently from Baseline (BL) to serum proteomics Endpoint (48 wks of ULT;EP), we found 21 and 49 significantly changed proteins (p < 0.05, Wilcoxon signed-ranks test) for Cohort 1 and 2, respectively. Interactome analysis through STRING-db, was accompanied by “pin-dropping” known gouty-inflammation markers, known to be below the mass spectrometry detection limits (30), along with the significantly altered proteins from both cohorts. We identified 23 high confidence interacting proteins (Fig. 2C), which Gene Ontology enrichment analyses revealed to belong to 4 major categories: Innate immune response, humoral immune response, protein/peptide secretion, and post-translation modification of proteins (Fig. 2D, Table 1).

There were 277 overlapping protein identifications between both independent cohorts. There were significant influences (PERMANOVA p < 0.10) from patient and timepoint on Cohort 1 and 2 results, respectively (Supplemental Fig. 1C&D). We subjected these proteins to interactome analysis, and observed 138 high confidence interacting proteins (Fig. 2E). Moreover, we identified 70 proteins that were similarly altered at 48wks ULT (Fig. 2E) in both cohorts. We also identified those proteins in our interactome that fell into innate or humoral gene ontology enrichment categories. Results showed rewiring of networked key inflammation mediators not detectable by conventional serum biomarker profiling, including C8 cleavage products, VIM, PPBP/CXCL7, KRT16, TGFB1, IGF-I, and sCD44. These novel biomarkers of XOI ULT effects were clustered with central gout mediators including IL-1B, CXCL8, IL6, and C5, in a tight protein interactome. Results revealed a novel functionally important network of physically interacting serum proteins in gouty inflammation that was altered in response to ULT to target with XOI drugs.

#### XOI treatment to serum urate target effects on the PBMC Proteome

**Figure 3:**
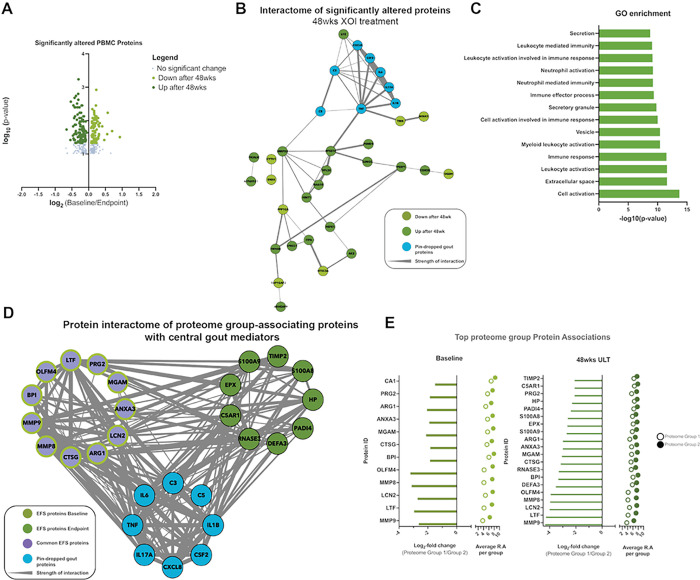
PBMC proteomics. A. Volcano plots of log2-fold change relative protein abundance versus log10 p-value. Points are colored by condition they are found higher in, and sized by p-value significance (p-value<0.05, Wilcoxon signed rank test). B. Protein interactome from String-DB using significantly altered proteins after ULT treatment of gout patients. Nodes are colored by group they are found to have higher relative abundance. C. Gene ontology enrichment analysis of significantly altered proteins after ULT. Enrichment was conducted on Cytoscape with the Human Proteome as background. D. PBMC patient proteome-associated protein abundances to understand PBMC patient proteome separation conducted at baseline and proteomics endpoint (48wks). E. Protein interactome from String-DB using top protein drivers of PBMC patient proteome separation along with “pin-dropped” central gout mediators. Nodes are colored by group they are found to have higher relative abundance.

Last, to further characterize *in vivo* response to XOI-based ULT in gout, we isolated PBMCs from Cohort 1 patients. We identified 197 significantly altered proteins at 48wks ULT (p < 0.05, Fig. 3A), with 42 high-confidence (> 0.700) interacting proteins (Fig. 3B) We found these proteins in the PBMC interactome (listed in Supplemental Table 2) belonging largely to secretion, leukocyte, and neutrophil activation gene ontology pathways (Fig. 3C). Moreover, the KRT protein findings for serum proteins were validated in the PBMC proteomics studies.

We next sought to understand how patient information associated to the PBMC proteome (Supplemental Fig. 2A) and found several cytokines to have significant influence (Supplemental Fig. 2C, p-value < 0.1). Interpatient correlation analysis identified two distinct proteome groups (Supplemental Fig. 2A-B), and statistical analysis identified proteins driving the separation of proteome group 1(n = 5) and 2 (n = 14). We analyzed samples separated by timepoint and identified the top scored proteins at Baseline and 48wks of ULT (Fig. 3D). We identified overlapping protein drivers of separation at both timepoints, and interactome analysis of identified driver proteins at both timepoints along with “pin-dropped” gout proteins (Fig. 3E) found strong and high confidence (> 0.700) interactions between known gout mediators and top identified proteins, particularly MMP9 and other proteins identified at 48wks ULT. Hence, PBMC proteome analysis further teased apart XOI-based ULT effects in gout patients while highlighting anti-inflammatory effects.

## Discussion

Gout requires a unique approach to arthritis targets and biomarkers of the response to XOI-based ULT, due to variable phenotypes, and weaving of urate homeostasis, comorbidities, and inflammatory arthritis (1–5, 8). In contrast to the genetics of urate biology, genome-wide association studies have identified few genetic coding variants potentially involved in gouty arthritis (31, 32). Therefore, this biomarker study assessed the biomarker potential of proteomic profiling of gout patient sera at 48wks sustained ULT to urate target with XOI that reduced both flare burden and serum urate in two independent cohorts.

Specific serum proteomics findings at 48wks XOI-based treat to target ULT, in both cohorts studied, included decreased C8A and C8G chains, which play a major role in complement C5b-9 MAC assembly and activity that, along with C5a generation, contribute substantially to the inflammatory process in model gouty arthritis (15, 16, 34). Paradoxically, we detected increase in serum of the NLRP3 inflammasome scaffolder and activation promoter VIM (vimentin)(35), of interest because early increase in gout flares is seen in XOI-based ULT (9), Increased serum sCD44 was noteworthy, since sCD44 inhibits macrophage phagocytosis of urate crystals and consequent NLRP3 inflammasome activation, by blocking crystal binding to transmembrane CD44 (36).

We also observed increase in serum of TGFB1, which promotes model gout flare resolution by suppressing macrophage activation by crystals (37). Conversely, IGF-I, which cross-talks with and can synergize with TGF-beta, was decreased in serum at 48wks ULT (38). We detected decrease in serum of the phagocyte-recruiting chemokine PPBP/CXCL7 (39), and decreased lactoferrin, a neutrophil-released co-activator of the lubricin-degrading serine protease Cathepsin G (40). That finding was of note, since Cathepsin G is a major degrader of lubricin, which functions as a substantial constitutive suppressor of gouty inflammation and urate production by synovial resident macrophages (41). We also observed an increase in monocyte/macrophage-expressed keratin-related proteins (KRT9,14,16), further validated by Cohort 1 gout patient PBMC proteomics. KRT16 is implicated in monocyte to macrophage differentiation, and MMP-1 and innate immune responses to tissue damage in epithelia (42).

Last, STRING-db analyses of significantly altered proteins from both cohorts revealed that the tight serum protein interactome network altered by XOI-based ULT encompassed a core group of central mediators of gouty inflammation (including IL-1B, CXCL8, IL6, C5)(4).

Robustness of our findings on effects of effective ULT on the serum protein interactome discovered here was buttressed by a group of parallel studies. First, in this context, previously published evidence in gout Cohort 1 that the ULT regimen altered the serum metabolome, and the serum lipidome in gout Cohorts 1 and 2, and effects of febuxostat on lipolysis in cultured adipocytes (25). Moreover, the current study demonstrated that the serum metabolome was significantly altered for purine and pyrimidine metabolism in Cohort 2, associated with significant changes in multiple other pathways, most pronounced for linoleate metabolism at both 24wks and 48wks ULT. Second, analyses of the Cohort 1 proteome of gout patient PBMCs identified 42 high-confidence interacting proteins belonging largely to secretion, leukocyte, and neutrophil activation gene ontology pathways. The KRT findings for serum proteins were validated in the PBMC proteome. In addition, we found strong and high confidence (> 0.700) interactions between known gout mediators and EFS identified proteins, particularly in the proteins identified at 48wks of ULT, including MMP9. Whereas no significant difference in MMP9 abundance levels was identified between BL and 48wks of ULT, further study would be needed to validate significance of differences between PBMC proteome groups 1 and 2. The collective results of PBMC proteome analysis further teased apart the effects of XOI-based ULT in gout, and highlighted anti-inflammatory effects of XOI-based ULT on these leukocytes as a whole.

We employed *in vitro* studies that characterized effects of the selective XOI febuxostat on the proteome of cultured murine BMDMs stimulated by the major gouty inflammation driver IL-1b. Febuxostat suppressed multiple pro-inflammatory IL-1b-induced changes in the macrophage proteome. Analyses of gene ontology enrichment of proteins found in the macrophage protein interactome revealed that *in vitro* XOI treatment of activated BMDMs broadly reversed many pro-inflammatory responses. Notably, the most pronounced pathway changes were seen in classical and alternative pathway complement activation, which reinforced the impact of the findings for XOI-treatment effects on C8A and C8G in the gout patient serum proteome. Febuxostat also altered lymphocyte-mediated immunity, fibrinolysis, and cytolysis gene ontology pathways in cultured macrophages in response to IL-1b. Our findings in cultured macrophages and gout patient PBMCs were novel partly because previous studies have suggested that both hyperuricemia and urate crystals program elevated monocyte inflammatory responses *in vitro* and that hyperuricemia primes model gout inflammation in mice *in vivo* model gout (43–45).

A pro-inflammatory serum proteome signature was recently characterized in asymptomatic hyperuricemia (AH) by targeted proteomics (46). The approach used the Olink Target 96 Inflammation Panel^™^ (46), distinct from the unbiased mass spectrometry-based approach utilized in the current study. The methodology employed dual recognition by oligonucleotide-labelled antibody probe pairs and DNA-coupled quantitative PCR, designed to detect specific immunoregulatory proteins below mass spectrometry detection limits (46). Upregulated serum immunoregulatory proteins in AH group included the mTOR effector 4E-BP1, IL-18R1, multiple growth factors, chemokines, members of the IL-6 cytokine and TNF superfamily, with a Th17 cell signature, and increases in inflammation-dampening IL-10 and FGF21 also identified (46). Using the same targeted serum proteomics approach, a small sub-study of 13 subjects before and 3 months into successful XOI-based treat to target ULT also revealed significant downregulation of LIF-R. CDCP1, IL-18, NT-3, IL10RB, CCL28, CCL11, and SLAMF1 (46). All of the differentially detected proteins in that targeted proteomics study, which were predominantly cytokines and growth factors, were below the detection limits of of our unbiased mass spectrometry serum proteomics approach (Sanchez, C, et al, unpublished observations). Therefore, the design, approach, and sample size of the current study were unique and provided distinct information on the molecular signature of XOI effects on hyperuricemia in gout.

Hyperuricemia increases blood monocyte population expansion in vivo in humans (44) However, monocytes, and other mononuclear leukocytes, are heterogeneous, and can be recruited into diseased or challenged tissues, and one limitation in this study is that monocytes are normally only a small fraction (ie, ≤ 10%) of PBMCs (47). PBMCs remain a source of highly informative biomarkers for acute and chronic inflammatory diseases, but also are highly heterogeneous (48), buttressing the limitation of this study that PBMCs only were obtained at the Cohort 1 site. This trial did not have a placebo or uricosuric treatment arm. Moreover, we did not study gout patient controls from the same clinical trial that failed to achieve serum urate target, However, the proportion of such subjects overall in the VA STOP GOUT trial was low (ie, ~ 20%)(19), and all those subjects were considered at least partially treated since they received XOI-based ULT.

In conclusion, a novel, functionally important network of physically interacting proteins in gouty inflammation was altered in response to sustained, effective XOI-based ULT. Potential clinical significance of the results, especially for data from the clinical trial, included that the treat to target XOI-based ULT regimen is associated with early increase in flare activity before gout flares eventually decrease (9). Moreover, the current study provides further support for the use of serum proteomics, including approaches targeting the complement pathway and the inflammatory secretome, to provide biomarkers for responses to gout pharmacotherapy, and for characterization and prognosis of different clinical phenotypes in the disease (41,46, 49, 50).

## Figures and Tables

**Table 1:** 

Cohort	Gene Abbreviation	Full Gene name	Function	Potential Role in Gouty Inflammation
**Cohort 1**	**GSN**	Gelsolin	Cytoskeletal protein	Unknown; previously found to be upregulated in serum of gout patients
	**IGF1**	Insulin-like growth factor I	Cell growth promotion	Antagonizes multiple TGFbeta responses
	**KRT**	KRT 9,14,16	Filament protein	Modulates monocyte to macrophage differentiation and connective tissue remodeling by MMP-1
	**LTF**	Lactotransferrin	Co-eleased from activated neutrophil granules with elastase with elastase and Cathepsin G proteases	C-activates Cathepsin G
	**SHBG**	Sex hormone-binding globulin	Receptor-mediated cell signaling	Suppresses inflammation in macrophages and adipocytes
	**IGLL5**	Immunoglobulin Lambda Like Polypeptide 5	Immunoglobulin	Modulation of inflammation
	**TGFBI**	Transforming growth factor beta 1	Modulates connective tissue homeostasis and infammation	Limits urate crystal induced inflammation, and rises in. resolution phase of model gouty inflammation
**Cohort 2**	**THBS1**	Thrombospondin-1	Abundant, ubiquitous cell adhesion protein	iIhibits neutrophil serine proteases (previously identified to be downregulated in patient sera in acute gout)
	**LCAT**	Phosphatidylcholinesterol acyltransferase	Central enzyme in the extracellular metabolism of plasma lipoproteins	Unknown
	**PPBP**	Platelet basic protein/CXCL7	Neutrophil-activating chemokine	Chemoattractant and activator of neutrophils
	**PROC**	Vitamin K-dependent protein C	glycoprotein	Unknown
	**CETP**	Cholesteryl ester transfer protein	Involved in the transfer of neutral lipids	Modulated lipoprotein metabolism
	**VIM**	Vimentin	Cytoskeletal protein	Activation-promoting scaffolding of the NLRP3 inflammasone
	**SPARCL1**	SPARC-like protein 1	Proliferation-Inducing Protein	Unknown
	**sCD44**	soluble CD44	Soluble from of the transmembrane signlaing receptor for hyaluronic acid and lubricin	Suppresses phagocytosis of MSU crystals and inflammation
	**PFN1**	Profilin-1	Binds to actin and affects the structure of the cytoskeleton	Unknown; implicated in rheumatoid arthritis
	**FCGR3A**	Fc Gamma Receptor IIIa	Antibody	Modulation of inflammation
**Gout-related**	**C3**	Central gout mediators		
	**C5**			
	**CSF2**			
	**CXCL8**			
	**IL17A**			
	**IL1B**			
	**IL6**			
	**TNF**			

## Data Availability

Raw proteomic and metabolomic data, as well as protein abundance tables can be accessed through massive.ucsd.edu via a MSV identifiers MSV000093638 (BMDMs) and MSV000093652 (Patient Serum).

## Abbreviations

AH: Asymptomatic hyperuricemia
BMDM: Bone marrow-derived macrophage
CDCP1: CUB domain-containing protein 1
EFS: Ensemble Feature Selection
FGF21: Fibroblast growth factor 21
IL10RB: IL-10 Receptor subunit beta
KRT: Keratin
LIF-R: Leukemia Inhibitory Factor Receptor
MMP: Matrix metalloproteinase
MSU: Monosodium urate
NT-3: Neurotrophin 3
NLRP3: NLR Family Pyrin Domain Containing 3
(PCA): Principal Component Analysis
(PCoA): Principal Coordinate Analysis
PBMC: Peripheral blood mononuclear cell
SLAMF1: Signaling Lymphocytic Activation Molecule Family Member 1
TMT: Tandem mass tag
ULT: Urate lowering therapy
VIM: Vimentin
XOI: Xanthine Oxidase Inhibition
